# Supplementary material for: A data-driven analysis of lumbar steroid injection satisfaction in patients with chronic low back pain
Source: Sci Rep. 2025 Jul 29;15:27734. doi: 10.1038/s41598-025-10907-0 (PMC12307618; doi:10.1038/s41598-025-10907-0)
Supplement: Supplementary file 1 — Supplementary Information. [file 41598_2025_10907_MOESM1_ESM.pdf]

Supplementary Material

Survey Measures and Response Options

This supplementary material provides a comprehensive overview of the survey measures and variables used in the study. It includes detailed descriptions of the questionnaires, response options, and scoring systems for the different assessed domains such as back pain assessment, medication-related perceptions, pain self-efficacy, motivation, and perceived care. The tables systematically present the structure of the questionnaires and their respective items, enabling a clear understanding of the data encoding for subsequent analysis.

Table 1. Core Outcome Measures Index (COMI) Components

Item & Question	Answer Options	Scoring
**Back Pain Intensity**		0-10
"How severe was your back pain in the last week?"	0 (No pain) to 10 (Worst pain)	
**Leg Pain Intensity**		0-10
"How severe was your leg/buttock pain in the last week?"	0 (No pain) to 10 (Worst pain)	
**Function**		0-10
"How much did your back problem interfere with normal work?"	Not at all<br>A little bit<br>Moderately<br>Quite a bit<br>Extremely	0<br>2.5<br>5.0<br>7.5<br>10.0
**Symptom-Specific Well-being**		0-10
"How would you feel spending the rest of your life with current symptoms?"	Very satisfied<br>Somewhat satisfied<br>Neither satisfied nor dissatisfied<br>Somewhat dissatisfied<br>Very dissatisfied	0<br>2.5<br>5.0<br>7.5<br>10.0
**Quality of Life**		0-10
"How would you rate your quality of life in the past week?"	Very good<br>Good<br>Moderate<br>Bad<br>Very bad	0<br>2.5<br>5.0<br>7.5<br>10.0
**Social Disability**		0-10
"In the past 4 weeks, how many days did you cut down on activities?"	None<br>1-7 days<br>8-14 days<br>15-21 days<br>>21 days	0<br>2.5<br>5.0<br>7.5<br>10.0
**Work Disability**		0-10
"In the past 4 weeks, how many days did back pain keep you from work?"	None<br>1-7 days<br>8-14 days<br>15-21 days<br>>21 days	0<br>2.5<br>5.0<br>7.5<br>10.0

Table 2. Back Pain History and Treatment Variables

Item & Question	Answer Options	Type
**Duration of Back Complaints**		Ordinal
"Since how many weeks are the complaints (again) so strong that you need treatment?"	Less than 4 weeks<br>4-8 weeks<br>8-12 weeks<br>More than 12 weeks	1<br>2<br>3<br>4
**Previous Infiltration**		Binary
"Have you had previous infiltrations?"	Yes<br>No	
**Time Since Last Infiltration**		Ordinal
"When was your last infiltration?"	Never<br>Less than 1 year<br>1-2 years<br>More than 2 years	
**Location of Last Infiltration**		Nominal
"Who performed your last infiltration?"	Never<br>Schulthess Clinic and same doctor<br>Schulthess Clinic from different doctor<br>Different clinic/institute	
**Previous Treatment Success**		Binary
"Did the previous infiltration help?"	Yes<br>No<br>Never (if no previous infiltration)	

Principal Features based on PCA

Principal Component Analysis of Features based on PCA

Principal Component Analysis revealed that 90% of the variance was in 23 components out of the total 52 encoded features. The ranking of the most informative features in terms of variance identified prior treatment experience and professional status as the most informative features (see supplementary material). Top features were previous infiltration improvement and professional occupation - housework, followed by doctor-recommended infiltration and care understanding. Clinical variables, such as baseline back pain, demonstrated moderate contributions, whereas psychosocial factors, including pain self-efficacy, had comparatively lower influence. Table 6 presents the features ordered by their summed loading values across principal components, with higher values indicating greater contribution to data variance.

Table 3. Medication-Related Variables

**Item & Question**	**Answer Options**	**Scoring**
**Susceptibility to Medications**	Likert Scale	Ordinal
"I am very susceptible to medications"	Strongly disagree<br>Disagree<br>Neutral<br>Agree<br>Strongly agree	1<br>2<br>3<br>4<br>5
**General Reaction to Medications**	Likert Scale	Ordinal
"I generally have strong reactions to medications."	Strongly disagree<br>Disagree<br>Neutral<br>Agree<br>Strongly agree	1<br>2<br>3<br>4<br>5
**Comparative Reaction Strength**	Likert Scale	Ordinal
"I generally react more strongly to medications than most people."	Strongly disagree<br>Disagree<br>Neutral<br>Agree<br>Strongly agree	1<br>2<br>3<br>4<br>5
**Experience of Side Effects**	Likert Scale	Ordinal
"I have experienced unpleasant side effects from medications in the past"	Strongly disagree<br>Disagree<br>Neutral<br>Agree<br>Strongly agree	1<br>2<br>3<br>4<br>5
**Dose Sensitivity**	Likert Scale	Ordinal
"Even a very small dose of medication can upset my body"	Strongly disagree<br>Disagree<br>Neutral<br>Agree<br>Strongly agree	1<br>2<br>3<br>4<br>5

Table 4. Pain Self-Efficacy Questionnaire (PSEQ)

Item & Question	Answer Options	Scoring
**Enjoyment Despite Pain**		1-6
"I can enjoy things despite the pain"	1 = Completely convinced<br>to<br>6 = Not at all convinced	
**Household Activities**		1-6
"I can do most household chores"	1 = Completely convinced<br>to<br>6 = Not at all convinced	
**Social Activities**		1-6
"I can meet friends/family<br>as often as before"	1 = Completely convinced<br>to<br>6 = Not at all convinced	
**Pain Coping**		1-6
"I can cope with pain in most situations"	1 = Completely convinced<br>to<br>6 = Not at all convinced	
**Work Capability**		1-6
"I can do some form of work despite pain"	1 = Completely convinced<br>to<br>6 = Not at all convinced	
**Leisure Activities**		1-6
"I can still do many enjoyable activities"	1 = Completely convinced<br>to<br>6 = Not at all convinced	
**Medication Independence**		1-6
"I can cope with pain without medication"	1 = Completely convinced<br>to<br>6 = Not at all convinced	
**Life Goals**		1-6
"I can still achieve most life goals despite pain"	1 = Completely convinced<br>to<br>6 = Not at all convinced	
**Normal Life**		1-6
"I can live a normal life despite the pain"	1 = Completely convinced<br>to<br>6 = Not at all convinced	
**Activity Progression**		1-6
"I can gradually become more<br>active despite pain"	1 = Completely convinced<br>to<br>6 = Not at all convinced	

Table 5. Motivation Sources and the care perception in Medical Decision-Making

**Item & Question**	**Answer Options**	**Type**
**Motivation Sources**	Checkbox	Categorical
	Motivation from friends	0
	Motivation from family	1
	Motivation from the infiltration doctor	2
	Motivation from the house doctor	3
	Motivation from the internet	4
	Motivation from own experience	5
**Importance of Others' Opinions**	Likert Scale	Ordinal
"How important is the opinion of others in your decision to get an infiltration?"	Not at all important	1
	Slightly important	2
	Moderately important	3
	Very important	4
	Extremely important	5
**Comfort with Care Received**	Likert Scale	Ordinal
"How comfortable do you feel with the care you received?"	Completely true	1
	Largely true	2
	Partially true	3
	Hardly true	4
	Not true at all	5
	Not assessable	6
**Understood by Healthcare Provider**	Likert Scale	Ordinal
"Do you feel understood by your healthcare provider?"	Completely true	1
	Largely true	2
	Partially true	3
	Hardly true	4
	Not true at all	5
	Not assessable	6
**Overall Experience with Care**	Likert Scale	Ordinal
"How positive is your overall experience with the care you received?"	Completely true	1
	Largely true	2
	Partially true	3
	Hardly true	4
	Not true at all	5
	Not assessable	6

Table 6. Feature Ranking Based on PCA Contribution

Rank	Feature	Summed loading	Rank	Feature	Value
1	Improved last infiltration (yes)	1.692	20	Improved last infiltration (never)	1.258
2	Profession (housework)	1.565	21	Motivation (friend)	1.256
3	Motivation (doctor)	1.532	22	Duration of back complaints	1.189
4	Last infiltration (different clinic)	1.519	23	Medicine reaction (than others)	1.181
5	Care understood	1.480	24	Previous infiltration	1.177
6	Care comfortability	1.441	25	Education	1.162
7	Profession (other)	1.401	26	Work disability	1.156
8	Medicine strong reaction	1.401	27	Medicine susceptibility	1.146
9	Motivation (own experience)	1.400	28	Profession (self-employed)	1.134
10	Work time	1.395	29	Age	1.132
11	Back pain	1.367	30	Pain self-efficacy (enjoy)	1.119
12	Profession (retired)	1.360	31	Time since last infiltration	1.057
13	Last infiltration (same clinic)	1.351	32	Function	1.052
14	Improved last infiltration (no)	1.340	33	Pain self-efficacy (no medicine)	1.026
15	Motivation (house doctor)	1.325	34	Motivation (family)	1.019
16	Last infiltration (different doctor)	1.311	35	Medicine weekly dose	1.012
17	Sex	1.297	36	Leg pain	0.978
18	Motivation (internet)	1.279	37	Pain self-efficacy (company)	0.754
19	Profession (incapacity)	1.263			
